# Evaluation des Projekts „TeleCOVID Hessen“ nach einjährigem Betrieb

**DOI:** 10.1007/s00101-023-01269-1

**Published:** 2023-03-17

**Authors:** Jenny Brandt, Michael Albert, Steffen Gramminger, Clemens A Greim, Nawid Khaladj, Cornelia Kolb, Ben Michael Risch, Michael Sander, Michael von Wagner, Kai Zacharowski, Ralf M. Muellenbach, Felix Hoffmann

**Affiliations:** 1 Universitätsmedizin der Johannes Gutenberg-Universität Mainz, Mainz, Deutschland; 2 Awesome Technologies Innovationslabor GmbH, Würzburg, Deutschland; 3 Hessische Krankenhausgesellschaft e. V., Eschborn am Taunus, Deutschland; 4 Klinik für Anästhesiologie, Intensiv- und Notfallmedizin, Klinikum Fulda gAG, Universitätsmedizin Marburg – Campus Fulda, Fulda, Deutschland; 5 Universitätsspital Zürich, Zürich, Deutschland; 6 Hessisches Ministerium für Soziales und Integration, Wiesbaden, Deutschland; 7 Universitätsklinikum Gießen und Marburg GmbH, Klinik für Anästhesiologie, operative Intensivmedizin und Schmerztherapie, Justus-Liebig-Universität Gießen, Gießen, Deutschland; 8 Stabsstelle Medizinische Informationssysteme und Digitalisierung, Universitätsklinikum Frankfurt, Frankfurt, Deutschland; 9 Medizinische Klinik 1, Universitätsklinikum Frankfurt, Frankfurt, Deutschland; 10 Klinik für Anästhesiologie, Intensivmedizin und Schmerztherapie, Universitätsklinikum Frankfurt, Goethe Universität, Frankfurt, Deutschland; 11 Klinik für Anästhesiologie, Intensivmedizin, Notfallmedizin und Schmerztherapie, Klinikum Kassel GmbH, Kassel, Deutschland; 12 Stabsstelle für medizinische Prozessentwicklung, Klinikum Darmstadt GmbH, Darmstadt, Deutschland; 13 APOLLON Hochschule der Gesundheitswirtschaft, Bremen, Deutschland

**Keywords:** COVID-19, Intensivmedizin, Telemedizin, Videosprechstunde, Pandemie, COVID-19, Intensive care, Telemedicine, Video consultation, Pandemic

## Abstract

**Hintergrund:**

Die hohe Fallzahl von intensivpflichtigen COVID-19-Patienten konfrontierte Krankenhäuser weltweit mit unerwarteten Herausforderungen. Dadurch gewann die strukturierte Vernetzung von Krankenhäusern eine besondere Bedeutung. Die teleintensivmedizinische Konsil- und Austauschplattform TeleCOVID wurde entwickelt, um die intensivmedizinische Versorgungsqualität zu verbessern.

**Fragestellung:**

Ziel der vorliegenden Studie war die Erhebung des Nutzungsverhaltens und der Nutzererfahrung der Anwender von TeleCOVID. Zusätzlich wurde untersucht, welche Implikationen sich für die Weiterentwicklung der telemedizinischen Anwendung ergeben.

**Material und Methoden:**

Im Mai 2022 wurde eine Anwenderbefragung mittels eines Online-Fragebogens durchgeführt. Die Distribution erfolgte über das Hessische Ministerium für Soziales und Integration (HMSI). Dabei wurden alle 135 Krankenhäuser Hessens per E‑Mail angeschrieben und zur Teilnahme an der Studie eingeladen.

**Ergebnisse:**

Die Studie hat gezeigt, dass TeleCOVID hauptsächlich für Verlegungsanfragen genutzt wurde, gefolgt vom Bedarf einer Therapiebesprechung ohne Verlegungsanfrage. Zudem zeigte sich, dass die Anwender besonders die Möglichkeit einer datenschutzkonformen und strukturierten Übermittlung von Patientendaten als vorteilhaft bewerten. Die fehlende Anbindung an das Krankenhausinformationssystem (KIS), der erhöhte Zeitaufwand durch den Log-in-Prozess sowie die eingeschränkte Primärerreichbarkeit der Ansprechpartner wurden kritisiert.

**Diskussion:**

Bei einer Weiterentwicklung der App sollten die Anbindung an das Krankenhausinformationssystem sowie eine Reduktion des Zeitaufwands bei der Anwendung erfolgen. Weiterhin sollten eine interoperable Dateninfrastruktur und die Einführung eines Webclient angestrebt werden. Eine Weiterführung und Ausweitung der App auf andere Indikationsbereiche ist empfehlenswert.

**Zusatzmaterial online:**

Die Online-Version dieses Beitrags (10.1007/s00101-023-01269-1) enthält den zugrunde liegenden Fragebogen.

## Abstract (en)

**Background:**

The SARS-CoV‑2 pandemic posed unexpected challenges for hospitals worldwide and in addition to the supply emergency, simultaneously caused a high pressure to innovate. Due to the high number of cases of COVID-19 patients requiring intensive care, structured networking of hospitals gained particular importance. The tele-ICU communication platform TeleCOVID was developed to improve the quality of intensive care both by enabling teleconsultations and by supporting patient transfers.

**Objective:**

The present study aimed to survey user experiences with TeleCOVID. The study investigated the extent to which the app is used, the user experiences of the participating hospitals, and the resulting implications for the further development of the telemedicine application.

**Material and methods:**

A user survey was conducted in May 2022 using an online questionnaire. The survey contained both closed and open questions with a free text field. It was sent via the Hessian Ministry of Social Affairs and Integration (HMSI). All 135 hospitals in Hesse were contacted by e‑mail and invited to participate in the study. The results of the closed questions were analyzed using descriptive statistics, and the results of the open questions were clustered and thematically summarized using qualitative content analysis.

**Results:**

The study showed that TeleCOVID was used primarily for transfer requests, followed by the need for a treatment consultation without a transfer request. Most often, ECMO treatment or treatment in a hospital of a higher care level was required. The content analysis showed that users particularly rated the possibility of a data protection-compliant and structured transfer of patient data as advantageous. It is also worth mentioning that in almost 25% of the cases a transfer of patients could be prevented by TeleCOVID. Disadvantages frequently mentioned by respondents were the lack of connection to the electronic hospital information system, the increased time required for the registration process, and the poor primary accessibility of contact persons.

**Conclusion:**

In a further development of the application the connection to the electronic hospital information system should be considered particularly urgent. In addition, the time expenditure should be reduced by a simplified login process. Due to interface barriers, an alternative data infrastructure would also be conceivable to create interoperability. The introduction of a web client could also increase usability. The main beneficiaries of hospital networking are physicians and patients in a context associated with a high workload and specific medical issues. Continuation and expansion of the app to intensive care medicine and beyond are therefore recommended. In further studies on the project, personal interviews with decision makers could be useful to conduct a more targeted needs analysis.

## Einführung zum Thema

Im Rahmen der Severe Acute Respiratory Syndrome Coronavirus Type 2(SARS-CoV‑2)-Pandemie waren Krankenhäuser innerhalb kurzer Zeit mit einer hohen Fallzahl an Patienten mit einer bis dahin weitestgehend unerforschten Erkrankung konfrontiert. Die telemedizinische Applikation „TeleCOVID Hessen“ (im Folgenden „TeleCOVID“) wurde entwickelt, um die intensivmedizinische Versorgungsqualität von Patienten in Hessen zu verbessern.

## Hintergrund und Fragestellung

Die SARS-CoV-2-Pandemie konfrontierte Krankenhäuser weltweit mit unerwarteten Herausforderungen und bewirkte neben dem Ausnahmezustand in der Versorgung gleichzeitig einen hohen Innovationsdruck [1].

Durch die hohe Fallzahl von intensivpflichtigen Coronavirus Disease 2019(COVID-19)-Patienten gewann die strukturierte Vernetzung von Krankenhäusern eine besondere Bedeutung, zum einen zur Bettenallokation bei der notwendigen Verlegung von Patienten und zum anderen zum datenschutzkonformen fachlichen Austausch bei akuter Verschlechterung des Gesundheitszustandes [2].

Telekonsile bzw. Videokonsile spielten im Rahmen der Pandemie eine große Rolle. Definiert werden diese durch die Kommunikation mittels digitaler Medien von mindestens zwei Ärzten, bei der es um die Vermittlung von Wissen und Kenntnissen über den gesundheitlichen Zustand eines Patienten geht [3].

Die TeleCOVID-Hessen-App wurde durch das Awesome Technologies Innovationslabor GmbH unter Beteiligung des Universitätsklinikums Frankfurt sowie des Klinikums Kassel aus einem Forschungsprojekt mit Schwerpunkt Wissenstransfer im Rahmen der Pandemie weiterentwickelt, um die intensivmedizinische Versorgungsqualität und die Ressourcenallokation im Rahmen der Pandemie zu verbessern. Beim Projekt TeleCOVID Hessen handelt es sich um ein Modellprojekt mit einer Anwendungserprobung. Die zugrunde liegende App AMP.clinic ist kein Medizinprodukt; das Unternehmen Awesome Technologies ist im Qualitätsmanagement für Medizinproduktehersteller nach ISO 13485 zertifiziert. Zur Nutzung der App wurden auf den hessischen Intensivstationen iPads (Firma Apple, Cupertino, CA, USA) bereitgestellt, die eigens zu diesem Zweck eingerichtet wurden, und auf denen keine darüber hinausgehenden Anwendungen möglich sind. Mithilfe der App ist ein datenschutzkonformer Austausch von medizinischen Informationen und Beratungen über Videokonsile möglich. TeleCOVID Hessen gewährleistet einen hohen Datenschutz. Nach Erhalt der mobilen Endgeräte ist eine Anmeldung eines in dem jeweiligen Krankenhaus tätigen Arztes notwendig. Danach erfolgt eine postalische Zustellung des Passwortes in die entsprechende Klinik, was einer doppelten Verifizierung der Zugangsberechtigung entspricht. Die Daten werden auf Servern der ekom21 – KGRZ Hessen, einem zertifizierten Rechenzentrum, gespeichert und automatisch nach 12 Monaten gelöscht [4].

Mittels einer Allgemeinverfügung vom 16.02.2021 wurde TeleCOVID für alle hessischen Krankenhäuser für die Verlegung von COVID-19-Patienten verpflichtend eingeführt und seitdem bis zur Evaluierung im Mai 2022 für etwa 400 Konsile genutzt. Die anfallenden Kosten für Hardware, Software sowie eine pauschale Aufwandsentschädigung wurden vom Land Hessen getragen [5–7]. Die App war zunächst dazu konzipiert worden, dass Anwender mithilfe des iPad Fotos von relevanten Patientendaten aufnehmen, den entsprechenden Kategorien wie Anamnese, Arztbrief, Blutgasanalyse, Radiologie oder Labor zuordnen, um dann im Rahmen eines Telekonsils einen gezielten und effektiven Austausch durchführen zu können. Während der Kommunikation kann durch die Gliederung nach Patienten die Vorgeschichte und der bisherige Informationsaustausch durch andere Kollegen nachvollzogen werden [4].

Im Rahmen dieser Studie wurde die Nutzererfahrung der Anwender von TeleCOVID systematisch erhoben, um die folgenden Forschungsfragen zu beantworten:

1. In welchem Umfang wurde TeleCOVID genutzt?
2. Wie ist die Nutzererfahrung in den teilnehmenden Krankenhäusern?
3. Welche Implikationen ergeben sich für die Weiterentwicklung von TeleCOVID?

## Studiendesign und Untersuchungsmethoden

**Figure 1:**
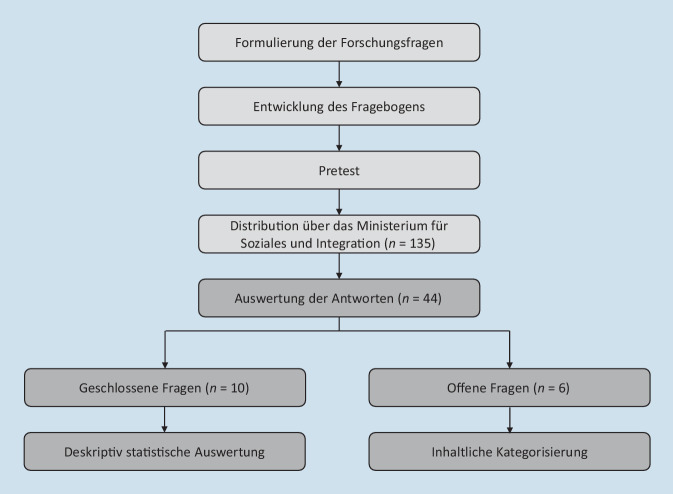

Das methodische Vorgehen wird in Abb. 1 grafisch dargestellt.

Zur Beantwortung der Forschungsfragen wurde eine Anwenderbefragung durchgeführt. Im Rahmen der Fragebogenentwicklung wurden entlang der Anwendungs-Journey Fragen konstruiert, um das Nutzungsverhalten und -erleben systematisch zu erfassen. Die Umfrage enthielt insgesamt 16 Fragen, darunter 10 geschlossene Fragen mit 2 bis 6 Antwortmöglichkeiten und 6 offene Fragen mit Freitextfeld. Bei den Fragen 2, 7 und 12 wurden die Teilnehmer bei der Antwort „Nein“ zu einer offenen Frage weitergeleitet, die bei der Antwort „Ja“ nicht gestellt wurde. Die Datenerhebung wurde mittels des Umfragedienstleisters Netigate durchgeführt.

Nach der Fragebogenkonstruktion erfolgte ein Pretest, der von Mitgliedern der TeleCOVID-Projektgruppe, die nicht an der Fragebogenerstellung beteiligt waren, durchgeführt wurde.

Am 16.05.2022 wurden über das Ministerium für Soziales und Integration (HMSI) alle 135 Krankenhäuser Hessens per E‑Mail angeschrieben und zur Teilnahme an der Studie eingeladen; hierzu wurde ein Link zu der Online-Befragung beigefügt [8]. Des Weiteren wurde am 17.05.2022 im Rundschreiben 357/2022 der Hessischen Krankenhausgesellschaft e. V. auf die Evaluierung von TeleCOVID aufmerksam gemacht [9]. Von den hessischen Krankenhäusern waren 78 Krankenhäuser in das TeleCOVID-Projekt eingebunden und haben die Anwendung somit potenziell genutzt. Die Befragung war vom 16.05.2022 bis zum 31.05.2022 geöffnet.

Die Ergebnisse der geschlossenen Fragen (Fragen 1 bis 7 und 11 bis 13) wurden mittels deskriptiver Statistik ausgewertet, die Ergebnisse der offenen Fragen (Fragen 8 bis 10 und 14 bis 16) wurden mittels qualitativer Inhaltsanalyse geclustert und thematisch zusammengefasst [10].

## Ergebnisse

Es haben 44 der angeschriebenen 135 Krankenhäuser (33 %) an der Studie teilgenommen. Vier teilnehmende Krankenhäuser haben die Befragung vorzeitig abgebrochen, von diesen wurden die beantworteten Fragen in die Auswertung einbezogen.

Nachfolgend wird die Auswertung der einzelnen Fragen beschrieben.

### Frage 1: Welche Kategorie trifft auf Ihr Krankenhaus zu?

**Figure 2:**
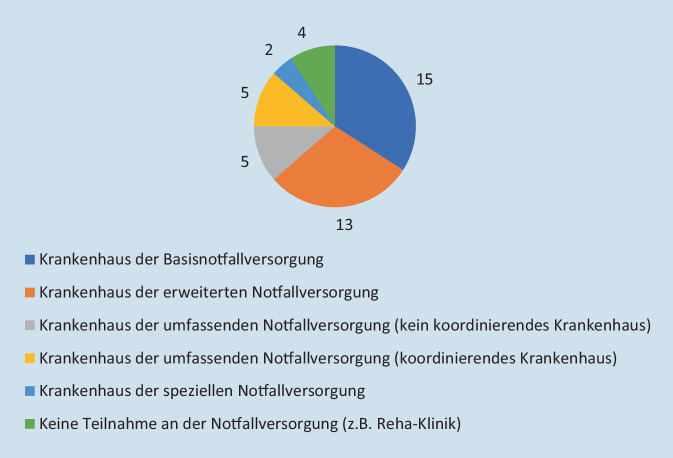

Zu dieser Frage liegen 44 Antworten vor (Abb. 2). Von den an der Umfrage teilnehmenden Krankenhäusern gehören 15 (34 %) zur Kategorie der Basisnotfallversorgung, 13 (30 %) zur erweiterten Notfallversorgung, 5 (11 %) zählen zur umfassenden Notfallversorgung, sind aber kein koordinierendes Krankenhaus, bei weiteren 5 (11 %) handelt es sich um Krankenhäuser der umfassenden Notfallversorgung als koordinierendes Krankenhaus, und 2 Krankenhäuser (5 %) gehören zur speziellen Notfallversorgung. Vier Krankenhäuser (9 %) nehmen nicht an der Notfallversorgung teil, weil es sich z. B. um Rehakliniken handelt.

### Frage 2: Haben Sie die TeleCOVID-Hessen-App genutzt?

Es liegen 43 Antworten vor. In 24 Krankenhäusern (56 %) wurde TeleCOVID genutzt; 19 Krankenhäuser (44 %) nutzen sie nicht.

Von den 19 Krankenhäusern, die die App nicht genutzt haben, nahmen 4 Krankenhäuser nicht an der Notfallversorgung teil, weil es sich beispielsweise um Rehakliniken handelt; sechs gehörten der Kategorie der Basisnotfallversorgung an, 5 der erweiterten Notfallversorgung, 2 der speziellen Notfallversorgung. Bei den Krankenhäusern der umfassenden Notfallversorgung nutzten 2 die App nicht, davon war ein Krankenhaus kein koordinierendes Krankenhaus, und ein koordinierendes Krankenhaus nutzte die App nicht.

### Frage 3: Wie häufig haben Sie die TeleCOVID-Hessen-App durchschnittlich geöffnet und/oder genutzt?

Es liegen 23 Antworten vor. Zu den Fragen 3 bis 12 wurden die Befragten nur weitergeleitet, wenn Frage 2 mit „Ja“ beantwortet wurde, weswegen diese Fragen nur von 23 Befragten beantwortet wurde.

Insgesamt 17 Krankenhäuser (74 %) nutzten die App weniger als einmal pro Monat. Sechs Krankenhäuser (26 %) nutzten die App ein- bis 5‑mal pro Monat. Kein Krankenhaus gab an, die App mehrmals wöchentlich oder täglich zu nutzen.

### Frage 4: Mit welchen Einrichtungen hatten Sie über die TeleCOVID-App Kontakt? (Mehrfachauswahl möglich)

Es liegen 22 Antworten vor. Von den Nutzern der App hatten 6 Krankenhäuser (27 %) Kontakt mit einem Krankenhaus der Basisnotfallversorgung, 4 Krankenhäuser (18 %) mit einem Krankenhaus der erweiterten Notfallversorgung, 4 Krankenhäuser (18 %) mit einem Krankenhaus der umfassenden Notfallversorgung, das kein koordinierendes Krankenhaus ist, 19 Krankenhäuser (86 %) mit einem koordinierenden Krankenhaus der umfassenden Notfallversorgung. Vier Krankenhäuser (18 %) hatten Kontakt mit einem Krankenhaus der speziellen Notfallversorgung; kein Krankenhaus hatte Kontakt mit einem Krankenhaus, das nicht an der Notfallversorgung teilnimmt (z. B. Reha-Klinik).

### Frage 5: Aus welchem Grund haben Sie die TeleCOVID-App am häufigsten genutzt? (Mehrfachauswahl möglich)

Es liegen 22 Antworten vor. In 15 Krankenhäusern (68 %) wurde TeleCOVID für eine Verlegungsanfrage an ein anderes Krankenhaus genutzt, acht Krankenhäuser (36 %) für eine Verlegungsanfrage von einem anderen Krankenhaus, und 7 Krankenhäuser (32 %) nutzten die App zur Therapiebesprechung ohne Verlegungsanfrage.

### Frage 6: Falls Sie Verlegungsanfragen an ein anderes Krankenhaus gerichtet haben, welche medizinischen Gründe lagen dem zugrunde? (Mehrfachauswahl möglich)

Es liegen 19 Antworten vor. Am häufigsten wurde mit 15 von 19 Antworten von den Befragten angegeben, dass eine ECMO-Therapie erforderlich gewesen sei (79 %), gefolgt mit 9 von 19 Antworten von der Notwendigkeit der Therapie in einem Krankenhaus einer höheren Versorgungsstufe (47 %). Sechsmal wurde Kapazitätsmangel von den Befragten angegeben (32 %). Zwei Krankenhäuser gaben andere Gründe an (11 %), und einmal wurde eine heimatnahe Rückverlagerung als medizinische Grundlage für die Verlegungsanfrage genannt (5 %).

### Frage 7: Hat die Kommunikation technisch störungsfrei funktioniert?

Es liegen 22 Antworten vor. Insgesamt 17-mal wurde mit „Ja“ geantwortet (77 %). Fünf Anwender beantworteten die Frage mit „Nein“ (23 %).

### Frage 7a: Falls Sie „Nein“ angegeben haben, welche Störungen sind aufgetreten?

Zu dieser Frage liegen 5 Antworten vor. Es lassen sich 2 Cluster voneinander abgrenzen:

1. *Technische Störungen:* Drei Antworten bezogen sich auf eine mangelnde Konnektivität oder Störungen der Kommunikation. Teilweise brauchte es mehrere Versuche, um eine erfolgreiche Verbindung herzustellen. Wenn Updates nicht hochgeladen worden waren, funktioniere die Übertragung nicht einwandfrei.
2. *Erreichbarkeit der Ansprechpartner:* Die übrigen 2 Antworten thematisieren Probleme bei der Erreichbarkeit der Ansprechpartner. So hatte in einem Fall das adressierte koordinierende Krankenhaus die Anfrage nicht erhalten. Diese erreichte ein anderes Krankenhaus im gleichen Ort; es gab hierzu jedoch keine Rückmeldung. Ein anderer Ansprechpartner konnte das Tablet nicht starten.

### Frage 8: Welche Vorteile hatte die Kommunikation gegenüber gängigen Kommunikationsmitteln (Telefon, Mobiltelefon, Fax)?

Es liegen 21 Antworten vor, aus denen sich 4 Cluster ergaben. Einige Antworten beziehen sich inhaltlich auf mehrere Punkte.

1. *Übermittlung von Patientendaten:* Hierbei bezogen sich die Befragten in 10 Fällen auf die Übermittlung von Laborwerten, Arztbriefen, Beatmungskurven und Bildmaterial, sowohl radiologischer Bildgebung als auch von Bildern vom Patienten selbst. Computertomographien seien jedoch nur als Screenshots übermittelbar. Positiv wurde die Sicherheit hinsichtlich des Datenschutzes gegenüber Mobiltelefonen bewertet. Auch die Möglichkeit einer vollständigen Befundübermittlung vor der persönlichen Kommunikation wurde als sinnvoll eingeschätzt.
2. *Übersichtlichkeit:* Diesem Cluster wurden 5 Antworten zugeordnet, die eine strukturierte Abfrage und Gliederung, eine koordinierte Zusammenfassung sowie den Verzicht auf das Ausdrucken von Fragebogen und Faxen nannten.
3. *Möglichkeit der Videokonsultation:* Von 3 Befragten wurde die Möglichkeit eines telemedizinischen Konsils genannt und die Kompetenz des Ansprechpartners positiv hervorgehoben.
4. *Keine Vorteile:* Acht Teilnehmer konnten keine Vorteile gegenüber der gängigen Kommunikation feststellen. Gründe dafür waren in erster Linie eine fehlende Anbindung an das Krankenhausinformationssystem (KIS), woraus viel Schreibarbeit resultiere. Die Nutzung der App wurde als zeitaufwendiger als ein Telefonat empfunden. Eine Anwendung auf dem PC wurde als wünschenswert beschrieben. Letztlich wurden die Bildqualität der Fotos bemängelt und der Fotografiervorgang mit dem Tablet als umständlich beschrieben.

### Frage 9: Welche Nachteile hatte die Kommunikation gegenüber gängigen Kommunikationsmitteln (Telefon, Mobiltelefon, Fax)?

Es liegen 22 Antworten vor, aus denen sich 7 Cluster ergaben. Fünf Antworten wurden nur einmalig genannt und waren inhaltlich keinem der anderen Cluster zuzuordnen. Daher wurden sie unter „Sonstiges“ zusammengefasst und im Folgenden näher erläutert. Einige Antworten beziehen sich inhaltlich auf mehrere Punkte.

1. *Zeitaufwand:* Der Zeitaufwand wurde in 8 Fällen als höher gegenüber gängigen Kommunikationsmitteln beschrieben. Dies habe besonders dann eine hohe Relevanz, wenn Patienten dringend verlegt werden müssen. Als Erklärung für den hohen Zeitaufwand wurde genannt, dass u. U. nicht alle Fragen, die als Pflichtfelder hinterlegt sind, in jeder Situation erforderlich seien. Da die Anfrage ohne Ausfüllen aller verpflichtenden Datenfelder nicht gestellt werden kann, führe dies dazu, dass die App seltener genutzt werde.
2. *Eingeschränkte Primärerreichbarkeit: *Die Nutzung der App müsse vorher telefonisch angekündigt werden, weil nicht sicher sei, ob der Ansprechpartner diese überhaupt nutzt bzw. regelmäßig auf Anfragen kontrolliert. Dieser Aspekt wurde in 6 Antworten genannt.
3. *Verfügbarkeit des Tablets:* Zwei Anwender bemängelten die Verfügbarkeit des Tablets. Teilweise werde das Tablet aus Sicherheitsgründen verschlossen, um es vor Diebstahl zu schützen, sodass es nachts oder im Dienst nicht verfügbar sei.
4. *Fehlendes WLAN: *In 2 Antworten wurde festgestellt, dass aufgrund von fehlendem oder eingeschränktem WLAN das Tablet nicht oder nicht überall im Krankenhaus genutzt werden könne.
5. *Kein Webclient:* Wiederum 2 Befragte erwähnten die Einführung der Anwendung als Webclient als wünschenswert, damit nicht zwischen PC und Tablet gewechselt werden müsse. Zudem falle damit die zusätzliche personalisierte Anmeldung auf dem Tablet weg, welche von mehreren Anwendern als aufwendig beschrieben wurde.
6. *Sonstiges:* Fünf Antworten wurden diesem Cluster zugeordnet. Von einem Anwender wurde genannt, dass es zu Übertragungsschwierigkeiten und technischen Problemen gekommen sei. Ein anderer Anwender beschrieb das Problem, dass es sich um ein neues Tool handele und daher für einige Mitarbeiter gewöhnungsbedürftig sei. Zudem wurde eine fehlende Anbindung ans Internet bemängelt. Als umständlich wurde empfunden, dass Daten nicht direkt, sondern nur als Foto übermittelt werden können, sowie die Tatsache, dass kein Stationszugang vorhanden sei, sondern jede Person sich mit eigenem Passwort individuell anmelden müsse.
7. *Keine Nachteile: *Zwei Anwender konnten keine Nachteile gegenüber der konventionellen Kommunikation feststellen.

### Frage 10: Haben Sie Verlegungen von COVID-Patient:innen über die TeleCOVID-Hessen-App organisiert?

Es liegen 22 Antworten vor. Zehn Befragte (45 %) haben die Verlegung von COVID-Patienten über TeleCOVID organisiert, und 12 Anwender (55 %) haben keine Verlegungen über die App organisiert.

### Frage 11: Konnte durch die Nutzung von TeleCOVID die Verlegung von Patient:innen verhindert werden?

Es liegen 22 Antworten vor. Fünf Krankenhäuser (23 %) gaben an, dass nach der Nutzung von TeleCOVID die Verlegung einzelner Patienten nicht mehr als erforderlich eingeschätzt wurde. In 17 Fällen (77 %) waren die Verlegungen auch nach der Nutzung von TeleCOVID immer erforderlich.

### Frage 12: Würden Sie die TeleCOVID-Hessen-App gerne weiterbenutzen?

Es liegen 22 Antworten vor. Elf Anwender gaben an, dass sie die App gerne weiternutzen würden, und wiederum 11 möchten die App nicht weiternutzen (jeweils 50 %).

### Frage 12a: Wenn NEIN: Was müsste sich verbessern, um eine Weiternutzung attraktiv zu machen?

Es wurden 9 Antworten gegeben, aus denen sich 5 inhaltliche Cluster ableiten lassen. Einige Antworten beziehen sich inhaltlich auf mehrere Punkte.

1. *Zeitaufwand minimieren:* Zur Minimierung des Zeitaufwands wurde vorgeschlagen, dass die App weniger Pflichtfelder enthalten solle. Zudem erschien es sinnvoll, die Anmeldung zu vereinfachen, indem man einen Stationszugang bereitstelle, sodass die individuelle Anmeldung mit Nutzernamen und Passwort wegfalle.
2. *Anbindung ans Krankenhausinformationssystem (KIS):* Die Nutzung werde attraktiver, wenn die Informationen aus dem bestehenden KIS direkt versendet werden können.
3. *Webclient einführen: *Die Nutzung wurde als umständlich empfunden, weil das Tablet oder auch das Ladekabel teilweise nicht auffindbar seien oder das Tablet aus Angst vor Diebstahl weggeschlossen sei. Zudem mangele es an der Akzeptanz der Mitarbeiter, wenn ein zusätzliches Device eingeführt werden müsse. Das Tablet habe keine zusätzlichen Funktionen, sodass die Einführung eines Webclient zur Nutzung mit dem PC sinnvoll erschien.
4. *Gewährleistung der Erreichbarkeit: *Als Vorschlag wurde eine Push-Funktion zum Diensttelefon genannt, da eine permanente Erreichbarkeit bei Anfrage von Konsilen gewährleistet werden solle.
5. *Weiternutzung von TeleCOVID ist nicht sinnvoll: *Unter diesem Punkt sind 3 Antworten zusammengefasst. Einmal wurde als Erklärung genannt, dass es kein WLAN gebe, sodass eine Nutzung nicht infrage käme, und 2 Antworten geben lediglich an, dass die App keine Vorteile biete.

### Frage 13: Was hat Sie davon abgehalten, TeleCOVID zu nutzen?

Zu dieser Frage wurden die Teilnehmer direkt weitergeleitet, wenn Frage 2 mit „Nein“ beantwortet wurde. Es liegen 17 Antworten vor, aus denen sich 7 Cluster ergeben. Einige Antworten beziehen sich inhaltlich auf mehrere Punkte.

1. *Zeitaufwand:* Diesem Cluster wurden 8 Antworten zugeordnet. Hier wurde genannt, dass eine persönliche Übergabe auf konventionellem Weg deutlich weniger Zeit in Anspruch nehme. Auch der aufwendige Log-in-Prozess wurde von mehreren Befragten bemängelt, da er für Ärzte im Tagesgeschäft zu kompliziert und zeitaufwendig sei, weshalb die App z. B. für eine Anfrage nach möglichen Intensivbetten als unbrauchbar eingeschätzt wurde. Auch in dieser Frage wurde der Wunsch nach „Sammel-Log-ins“ bzw. einem Stationszugang genannt.
2. *Keine Notwendigkeit:* Fünf der Befragten gaben an, dass keine Notwendigkeit für die Nutzung vorliege. Entweder bestand kein Konsilbedarf, keine Teilnahme an der Notfallversorgung, das Haus habe keine Intensivmedizin oder versorge keine COVID-Patienten, weil es sich z. B. um ein psychosomatisches Krankenhaus handele.
3. *Fehlende KIS-Anbindung:* Drei Befragte gaben an, dass sie die App nicht genutzt hätten, weil eine Anbindung an das Krankenhausinformationssystem fehle.
4. *Keine Verfügbarkeit des Gerätes:* Zwei Befragte gaben an, dass das Gerät nicht verfügbar gewesen sei. Ein Anwender beschrieb, dass eine Freigabe der Geräte durch die IT-Abteilung erst ab Oktober 2021 erfolgte; das Tool sei danach im Testbetrieb „nicht mehr tragbar“ gewesen. Ein anderer Anwender gab an, dass keine Übermittlung von Anmeldedaten stattgefunden habe.
5. *Technische Gründe:* Drei Befragte gaben an, dass es kein funktionierendes Internet auf der Intensivstation oder generell im Krankenhaus gebe, bzw. dass die App von der Firewall des „freien WLAN“ blockiert werde.
6. *Sonstiges: *Unter diesem Cluster sind 3 Antworten zusammengefasst. Zum einen war der Grund für die Nichtnutzung die fehlende Vernetzung mit anderen Kinderintensivstationen, zum anderen die Tatsache, dass primärer Kooperationspartner ein anderes Bundesland sei. Die fehlende Akzeptanz in anderen Häusern wurde von einem Anwender als Grund dafür genannt, dass die App nicht genutzt worden sei.

### Frage 14: Was müsste sich verbessern, um eine Nutzung künftig attraktiv zu machen?

Auch zu dieser Frage mit Freitextfeld wurden Teilnehmer nur dann geleitet, wenn sie die App nicht genutzt hatten. Es liegen 16 Antworten vor, aus denen 4 inhaltliche Cluster zusammengefasst wurden. Einige Antworten beziehen sich inhaltlich auf mehrere Punkte.

1. *Anbindung ans KIS:* Von 5 der Befragten wurde genannt, dass eine Anbindung an das jeweilige Krankenhausinformationssystem stattfinden müsse, um die Nutzung künftig attraktiver zu machen. Zudem solle „die jeweilige IT in den Prozess involviert werden“.
2. *Zeitaufwand:* Der Zeitaufwand müsse minimiert werden. In 4 Antworten wurde von den Befragten gefordert, dass der Anmelde- bzw. Einwahlprozess vereinfacht werden müsse, z. B. durch „Sammel-Log-ins“.
3. *Keine konkreten Verbesserungsvorschläge:* Vier Anwender gaben keine konkreten Verbesserungswünsche an, oder die Antwort war nicht auswertbar.
4. *Sonstiges:* Bei speziellen Konstellationen sei die App nicht nutzbar (z. B. ein Haus an der Landesgrenze oder eine Kinderklinik). Somit ergibt sich der Verbesserungswunsch, dass zum einen die App auch anderen Bundesländern zugänglich gemacht werden solle, und zum anderen, dass die Nutzung auch Kinderkliniken einschließen solle. Ein Anwender schrieb: „Nicht Krankenhäuser sollten angefragt werden, sondern Ärzte mit ausgewiesener intensivmedizinischer Expertise“. Des Weiteren wurde von 2 Befragten die Forderung nach einer Vereinheitlichung des Datenschutzes gestellt. Ein Anwender schlug vor, Verantwortlichkeiten und Vergütung zu klären.

## Diskussion

Die telemedizinische Vernetzung von Intensivstationen wurde im Rahmen einer Studie von Lebreton et al. zum Einsatz der Extrakorporalen Membranoxygenierung (ECMO) bei Patienten mit SARS CoV-2-Infektion bereits als positiv bewertet [11]. Zudem zeigen Dohmen et al. in ihrer Arbeit einen messbaren Nutzen für COVID-19-Patienten durch die Etablierung eines intensivmedizinischen Versorgungsnetzwerkes in Nordrhein-Westfalen [12]. In dieser Studie sollte untersucht werden, inwieweit TeleCOVID ebenfalls einen Nutzen entfaltet und welche Verbesserungen künftig vorgenommen werden müssten, um die weitere Nutzung attraktiver und zielführender zu gestalten.

Die Befragung hat ergeben, dass die Anwender die Möglichkeit einer datenschutzkonformen strukturierten Übermittlung von Patientendaten sowie eine Videokonsultation grundsätzlich als vorteilhaft bewerten. Knapp 25 % der Befragten gaben an, dass eine u. U. für den Patienten belastende Verlegung von Patienten nach der Nutzung von TeleCOVID nicht mehr erforderlich waren.

Von den 44 Teilnehmern der Befragung nutzten 24 die App, und von diesen wurde sie ein- bis 5‑mal/Monat oder seltener genutzt. Zudem ergab die Befragung, dass genau die Hälfte der Befragten die App weiternutzen will.

Die Nachteile, die von den Befragten in der inhaltlichen Analyse gehäuft angegeben wurden, waren die fehlende Anbindung an das Krankenhausinformationssystem (KIS), der erhöhte Zeitaufwand durch den Log-in-Prozess und zu viele Pflichtfelder beim Ausfüllen der Anfrage, aber auch die schlechte Primärerreichbarkeit der Ansprechpartner. Aus diesen Punkten ergeben sich bereits die Empfehlungen, die bei einer Weiterentwicklung der App als besonders dringlich angesehen werden sollten, um die Nutzung attraktiver zu gestalten.

### Implikationen für die Weiterentwicklung von TeleCOVID

Von einer Vernetzung der Intensivstationen können nicht nur Patienten und Ärzte im Rahmen der COVID-19-Behandlung profitieren, sondern potenziell auch Akteure im Gesundheitswesen im Rahmen anderer Indikationen. Eine Ausweitung des Anwendungsspektrums auf weitere Indikationen (z. B. Kinderkliniken, Spezialambulanzen, onkologische Zentren, etc.) ist daher empfehlenswert. Dabei sollten die hier festgestellten Hürden bestmöglich berücksichtigt werden, um eine erfolgreiche Implementierung und Nutzung zu gewährleisten.

Bei der Weiterentwicklung von TeleCOVID sollte eine Anbindung an das Krankenhausinformationssystem (KIS) erfolgen, und der Zeitaufwand sollte durch einen vereinfachten Log-in-Prozess reduziert werden. Allerdings ist der Log-in-Prozess bereits jetzt schon personalisiert und mit einem 6‑stelligen Code recht simpel gehalten. Ein Gruppen- oder Stations-Log-in bringt datenschutztechnische Hürden mit sich und lässt sich nicht ohne Weiteres umsetzen.

Die Einführung eines Webclient könnte einerseits die Akzeptanz der Anwender erhöhen, weil das Tablet als zusätzliches Device wegfällt, und zum anderen auch das Problem lösen, dass in einigen Krankenhäusern kein flächendeckendes WLAN verfügbar ist. Nach Abschluss der Befragung wurde ein Webclient etabliert. Push-Mitteilungen werden Ende 2022 verfügbar sein, sodass die Erreichbarkeit bei Anfrage von Konsilen deutlich verbessert wird.

Aufgrund von Schnittstellenbarrieren wäre zur Sicherstellung der Interoperabilität auch die Anbindung an die existierende Dateninfrastruktur über data repositories denkbar, in dem die Gesundheitsdaten aus verschiedenen Anwendungen zentral abgelegt und somit ubiquitär verfügbar werden [13].

Eine Limitierung der bestehenden Nutzung besteht darin, dass TeleCOVID lediglich hessenweit implementiert wurde und über die Landesgrenzen hinaus nicht angewendet werden konnte. Um den katastrophenmedizinischen Anforderungen wie der länderübergreifenden Kleeblattkoordination oder dem in Hessen bereits etablierten IVENA-System gerecht zu werden, ist eine möglichst schnittstellenoffene Gestaltung der Software essenziell, um eine anbieterunabhängige Kommunikation zu ermöglichen.

### Limitationen

Limitierend für diese Arbeit ist die Teilnehmerzahl von 44 Befragten. Zudem muss angemerkt werden, dass von den Teilnehmern, welche die App nutzten, die Häufigkeit auf ein- bis 5‑mal/Monat oder sogar weniger als einmal/Monat begrenzt war. Somit lassen sich die Ergebnisse nicht unmittelbar auf die gesamte Nutzergruppe übertragen, und es fehlen u. U. einige Details. Eine Verzerrung der Teilnehmerzahl ist möglich, wenn aus einem Krankenhaus mehrere Personen teilgenommen haben, was aufgrund der Anonymisierung der Umfrage nicht auszuschließen ist.

Insgesamt geben die Antworten trotz der Limitationen wichtige Hinweise auf Verbesserungspotenzial und sollten für die Weiterentwicklung des Projekts genutzt werden.

## Fazit für die Praxis

- Die telemedizinische Vernetzung von Krankenhäusern ist dazu geeignet, die Versorgungsqualität zu verbessern. Für eine dauerhafte Implementierung von TeleCOVID müssen jedoch noch einige Hürden ausgeräumt werden.
- Bei der Befragung wurde ein standardisierter Fragebogen verwendet. In folgenden Studien zur Planung der weiteren Umsetzung des Projekts könnten persönliche Interviews mit Entscheidungsträgern sinnvoll sein, um eine gezieltere Bedarfsanalyse durchzuführen und Informationen zu erlangen, die in dieser standardisierten Form der Befragung nicht erfasst werden konnten.
- Im Vordergrund von Digitalisierungsprojekten darf nicht die Elektrifizierung bestehender Prozesse stehen, sondern der Aufbau und die digitale Unterstützung sinnvoller Versorgungsprozesse.

## Supplementary Information




