# Supplementary material for: Evaluation of the “TeleCOVID Hesse” project after 1 year in operation
Source: Anaesthesiologie. 2023 Mar 17;72(5):317–24. [Article in German] doi: 10.1007/s00101-023-01269-1 (PMC10022556; doi:10.1007/s00101-023-01269-1)
Supplement: Supplementary file 1 [file 101_2023_1269_MOESM1_ESM.pdf]

Zusatzmaterial zum Beitrag „Evaluation des Projektes „TeleCOVID Hessen“ nach einjährigem Betrieb“ von Brandt J, Albert M, Gramminger S et al. (2023) in *Die Anaesthesiologie*

Beitrag und Zusatzmaterial stehen Ihnen auf www.springermedizin.de zur Verfügung. Bitte geben Sie dort den Beitragstitel in die Suche ein.

Fragebogen zur Studie

Einleitungsfragen

Welche Kategorie trifft auf Ihr Krankenhaus zu?

- Krankenhaus der Basisnotfallversorgung
- Krankenhaus der erweiterten Notfallversorgung
- Krankenhaus der umfassenden Notfallversorgung (Kein koordinierendes Krankenhaus)
- Krankenhaus der umfassenden Notfallversorgung (Koordinierendes Krankenhaus)
- Krankenhaus der speziellen Notfallversorgung
- Keine Teilnahme an der Notfallversorgung (z.B. Reha-Klinik)

Haben Sie die TeleCOVID Hessen App genutzt?

- JA → Weiter mit Fragebogen 1
- NEIN → Weiter mit Fragebogen 2

Fragebogen 1

Wie häufig haben Sie die TeleCOVID Hessen App durchschnittlich geöffnet und/oder genutzt?

- Täglich
- Mehrmals wöchentlich
- 1 bis 5x pro Monat
- Weniger als 1x pro Monat

Mit welchen Einrichtungen hatten Sie über die TeleCOVID-App Kontakt? (Mehrfachauswahl möglich)

- Krankenhaus der Basisnotfallversorgung
- Krankenhaus der erweiterten Notfallversorgung

- Krankenhaus der umfassenden Notfallversorgung (Kein koordinierendes Krankenhaus)
- Krankenhaus der umfassenden Notfallversorgung (Koordinierendes Krankenhaus)
- Krankenhaus der speziellen Notfallversorgung
- Keine Teilnahme an der Notfallversorgung (z.B. Reha-Klinik)

Aus welchem Grund haben Sie die TeleCOVID-App am häufigsten genutzt?

- Verlegungsanfrage an anderes Krankenhaus
- Verlegungsanfrage von anderem Krankenhaus
- Therapiebesprechung ohne Verlegungsanfrage

Falls Sie Verlegungsanfragen an ein anderes Krankenhaus gerichtet haben, welche medizinischen Gründe lagen dem zugrunde?

- Therapie in Krankenhaus einer höheren Versorgungsstufe erforderlich
- Heimatnahe Rückverlegung
- ECMO-Therapie erforderlich
- Kapazitätsmangel
- Andere Gründe

Hat die Kommunikation technisch störungsfrei funktioniert?

- Ja
- Nein

Falls Sie „Nein“ angegeben haben, welche Störungen sind aufgetreten?

Freitextfeld

Welche Vorteile hatte die Kommunikation gegenüber gängigen Kommunikationsmitteln (Telefon, Mobiltelefon, Fax)?

Freitextfeld

Welche Nachteile hatte die Kommunikation gegenüber gängigen Kommunikationsmitteln (Telefon, Mobiltelefon, Fax)?

Freitextfeld

Haben Sie Verlegungen von Covid-Patienten über die TeleCOVID Hessen App organisiert?

- Ja
- Nein

Konnte durch die Nutzung von TeleCOVID die Verlegung Patient:innen verhindert werden?

- Ja
- Nein

Würden Sie die TeleCOVID Hessen App gerne weiter benutzen?

- Ja
- Nein

Wenn NEIN: Was müsste sich verbessern, um eine Weiternutzung attraktiv zu machen?

Freitextfeld

Danke für die Teilnahme.

Fragebogen 2

Was hat Sie davon abgehalten, TeleCOVID zu nutzen?

Freitextfeld

Was müsste sich verbessern, um eine Nutzung künftig attraktiv zu machen?

Freitextfeld

Danke für die Teilnahme.
